# Acute torrential mitral regurgitation during transcatheter aortic valve replacement: a case report

**DOI:** 10.1186/s40792-018-0446-z

**Published:** 2018-04-18

**Authors:** Yoshiyuki Yamashita, Hiromichi Sonoda, Tomoki Ushijima, Akira Shiose

**Affiliations:** Department of Cardiovascular Surgery, Kyushu University Hospital, 3-1-1 Maidashi, Higashi-ku, Fukuoka, 812-8582 Japan

**Keywords:** Transcatheter aortic valve implantation, Ischemic mitral regurgitation, Conduction disturbance

## Abstract

**Background:**

Transcatheter aortic valve replacement (TAVR) is a minimally invasive approach to aortic valve replacement. However, critical cardiovascular collapse can occur during the procedure for various reasons.

**Case presentation:**

A 90-year-old man with severe aortic stenosis and left circumflex artery stenosis developed acute torrential mitral regurgitation (MR) during TAVR. The valve deployment process induced left ventricular dyssynchrony due to left bundle-branch block and myocardial ischemia in the left circumflex artery region with torrential MR. Transesophageal echocardiography clearly demonstrated the mechanisms of MR, which was successfully bailed out by left ventricular pacing and intra-aortic balloon pumping.

**Conclusions:**

MR can be seriously exaggerated by various and complicated mechanisms during TAVR and should be rapidly assessed and appropriately managed depending on its mechanisms.

**Electronic supplementary material:**

The online version of this article (10.1186/s40792-018-0446-z) contains supplementary material, which is available to authorized users.

## Background

Transcatheter aortic valve replacement (TAVR) is rapidly becoming the standard of care for the treatment of aortic stenosis (AS) for patients with an increased surgical risk because of its minimal invasiveness and good short- and mid-term outcomes [1]. However, critical hemodynamic collapse can sometimes occur during TAVR. We herein describe a case of acute mitral regurgitation (MR) during TAVR induced by complicated mechanisms: left ventricular (LV) dyssynchrony due to left bundle-branch block (LBBB) and myocardial ischemia with papillary muscle dysfunction.

## Case presentation

**Fig. 1:**
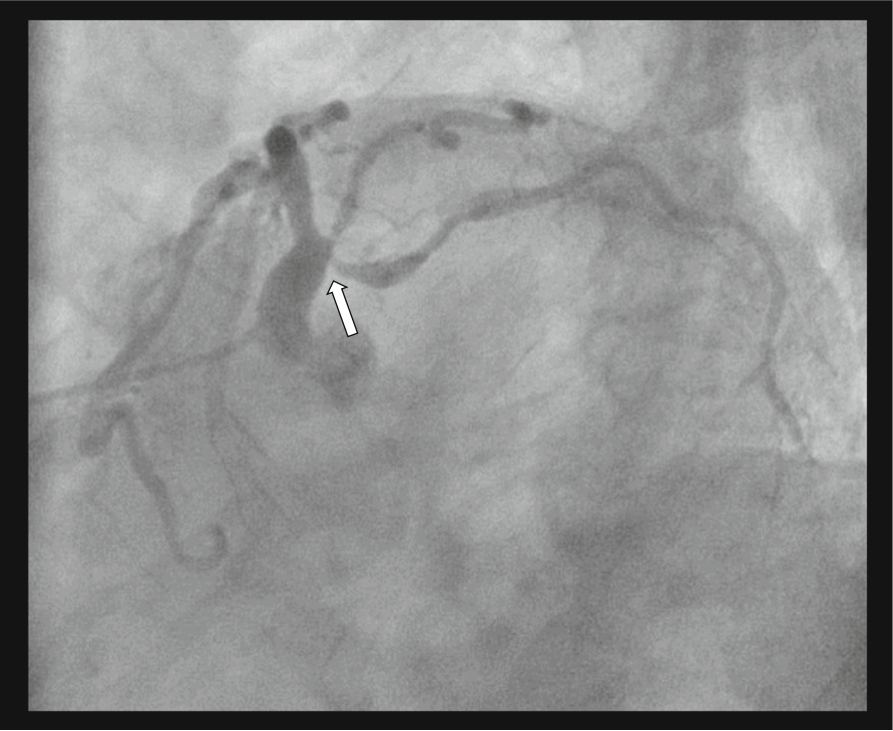
Preoperative coronary angiography findings. Left anterior oblique-caudal view showing severe ostial stenosis of the left circumflex artery

A 90-year-old man was referred to our hospital because of symptomatic severe AS. Six years previously, he had undergone endovascular aortic repair for an abdominal aortic aneurysm. Preoperative electrocardiogram showed normal sinus rhythm without abnormal Q waves or bundle-branch block (QRS duration of 106 ms). Echocardiography confirmed severe AS with a valve area of 0.58 cm^2^, peak velocity of 4.1 m/s, preserved LV ejection fraction of 54% without local asynergy, and mild MR without prolapse or annular dilatation. During cardiac catheterization, both the pulmonary capillary wedge pressure and pulmonary arterial pressure (PAP) were within the normal limits. Coronary angiography showed 90% stenosis of the ostial left circumflex artery (LCX) (Fig. 1). Because the LCX was relatively small and the patient had no symptoms of angina or local asynergy in the LV wall motion, we determined that prior or concomitant coronary intervention was unnecessary. Computed tomography revealed an aortic arch aneurysm and the implanted stent-graft in the abdominal aorta. Because of the patient’s advanced age, high surgical risks (Society of Thoracic Surgery score of 18.6%, logistic EuroSCORE of 27.5%), and presence of aortic lesions, transapical TAVR was planned.

**Fig. 2:**
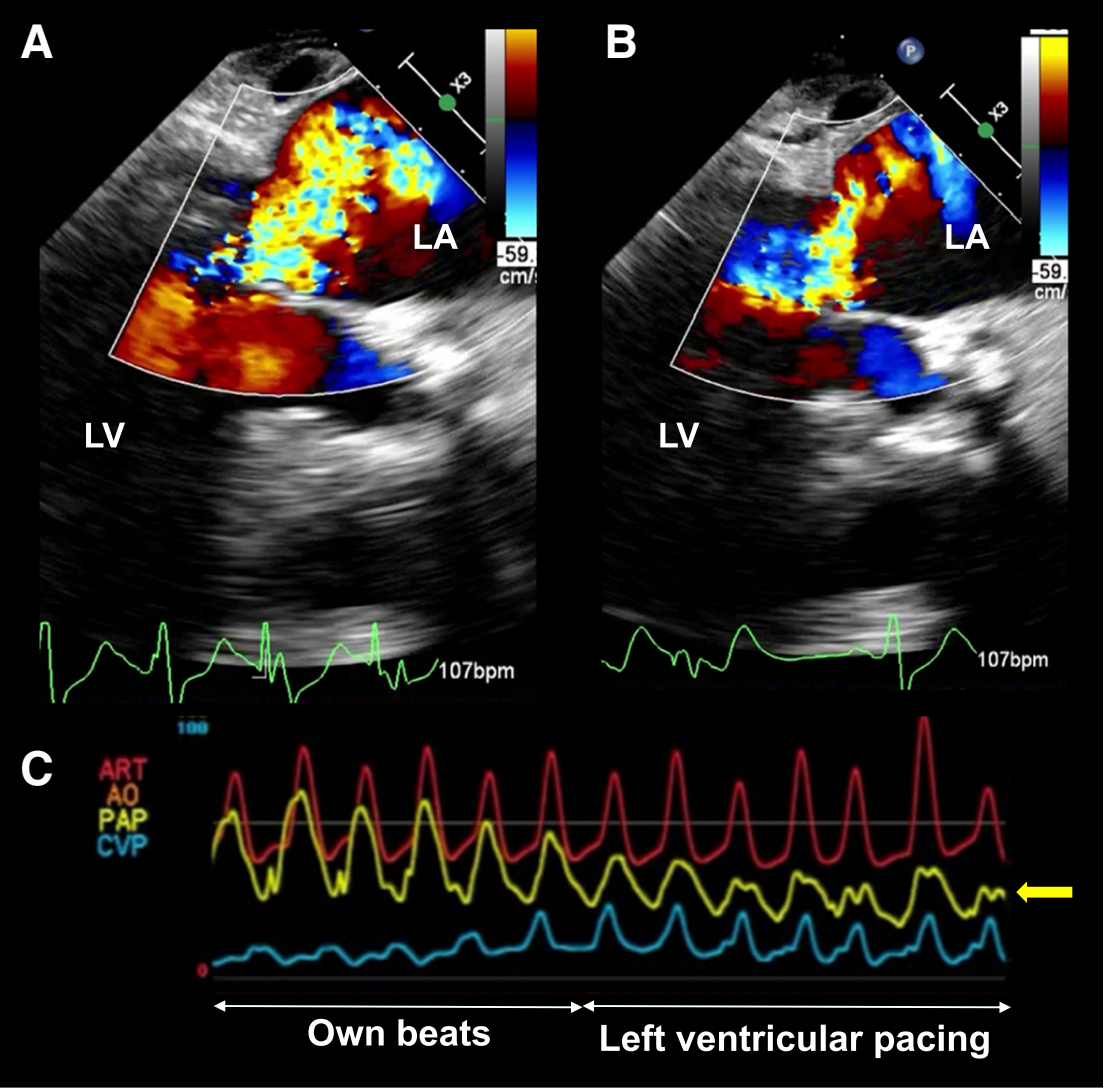
Intraoperative findings. Transesophageal echocardiography showing severe mitral regurgitation (**a**), which improved by left ventricular pacing (**b**). Monitoring results showing the decrease in pulmonary arterial pressure with left ventricular pacing (**c**). The yellow line (arrow) indicates the pulmonary arterial pressure. LA left atrium, LV left ventricle


Under general anesthesia, the apex was exposed via left anterolateral thoracotomy at the fifth intercostal space. After an extra-stiff guidewire had been passed through the aortic valve via the apex, the patient’s hemodynamic state slightly deteriorated because of new-onset LBBB with moderate MR and elevated PAP (systolic systemic arterial pressure (sSAP), 84 mmHg; systolic PAP (sPAP), 38 mmHg). Repositioning of the extra-stiff guidewire failed to relieve the LBBB, but the patient’s hemodynamic state was stable enough to continue the procedure. The delivery system was advanced through the aortic valve, and a 26-mm Sapien XT valve (Edwards Lifesciences, Irvine, CA) was successfully deployed under brief rapid ventricular pacing (RVP). After deployment, however, blood pressure recovery was poor and required brief chest compression and adrenalin injection. Although the patient’s blood pressure gradually increased, marked pulmonary hypertension (sSAP, 74 mmHg; sPAP, 60 mmHg) was noted. Transesophageal echocardiography (TEE) showed noncoapting mitral valve leaflets with torrential MR but an appropriately positioned prosthetic valve without significant paravalvular leakage, systolic anterior motion of the mitral valve leaflet, or pericardial effusion. After the removal of the delivery system from the apex, TEE revealed not only LV dyssynchrony due to LBBB but also relative hypokinetic wall motion in the posterolateral region with remarkable mitral posterior leaflet tethering, implying ischemic MR (Additional file 1: Video S1). Coronary angiography showed an unchanged coronary tree compared with the preoperative one: no evidence of coronary occlusion or slow coronary flow was present despite the originally stenosed LCX. We judged that in addition to LV dyssynchrony, relative myocardial ischemia in the LCX region (induced by RVP and subsequent persistent hypotension) had caused posterior papillary muscle dysfunction and acute severe MR. LV pacing for dyssynchrony was immediately employed using a myocardial electrode directly connected to the exposed left ventricle. The LV pacing was effective, and both the MR and PAP significantly decreased (Fig. 2); however, these changes were not enough to stabilize the patient’s hemodynamic state. Intra-aortic balloon pump (IABP) support was subsequently initiated while continuing LV pacing. Several minutes after initiation of IABP support, the posterior leaflet tethering and LBBB improved, leading to fair coaptation of the mitral leaflets. As a result, the MR drastically improved to the baseline level (Additional file 2: Video S2) and the patient’s hemodynamic state was stabilized (sSAP, 115 mmHg; sPAP, 26 mmHg). The IABP was removed on the first postoperative day, and the postoperative course was almost uneventful. Postoperative echocardiography showed good prosthetic valve function with mild MR.

Additional file 1:**Video S1.** Intraoperative transesophageal findings just after deployment of a transcatheter heart valve. Transesophageal echocardiography showing severe tethering of posterior mitral leaflet resulting in coaptation loss of the mitral valve leaflets with severe mitral regurgitation. (WMV 16455 kb)
Additional file 2:**Video S2.** Intraoperative transesophageal findings after initiation of intra-aortic valve pumping. Transesophageal echocardiography showing regained coaptation of the mitral valve leaflets and mild mitral regurgitation. (WMV 6267 kb)


TAVR has been established as an alternative to surgical aortic valve replacement for appropriately selected patients with severe symptomatic AS because of its minimal invasiveness [1]. However, cardiovascular collapse during TAVR can occur for various reasons. Acute MR is one serious complication that can cause cardiogenic shock and/or pulmonary edema. MR can become exaggerated during TAVR for several reasons, including direct mechanical damage to the mitral valve leaflets or subvalvular apparatus by the guidewire or prosthetic valve; secondary mechanisms include new-onset LBBB, myocardial ischemia, systolic anterior motion of the mitral valve leaflet, significant paravalvular leakage, and cardiac tamponade [2]. Therefore, rapid diagnosis and appropriate management according to the precise mechanisms are mandatory. Although TAVR is increasingly performed using a “minimalist approach” under local anesthesia without TEE monitoring for selected patients [3], many authors have reported the superiority of using TEE compared with transthoracic echocardiography to detect the degree and mechanism of new or worsening MR [2, 4]. In the present case, severe MR caused by complicated mechanisms (LV dyssynchrony and myocardial ischemia with papillary muscle dysfunction) were clearly detected by TEE. The possible causes of new-onset mitral valve leaflet tethering during TAVR include myocardial ischemia and deformity of the chordae or papillary muscle caused by the delivery system. Mechanical damage can also be detected by TEE with specific findings such as wire impingement of the chordae [5]; however, determining an accurate differential diagnosis from myocardial ischemia can be difficult. Therefore, in the present case, after rapid assessment of good prosthetic valve function, we removed the delivery system to exclude mechanical damage to the mitral valve complex and repeatedly examined the degree and mechanism of MR. Persistent tethering of the mitral leaflets confirmed that myocardial ischemia had induced the tethering. In general, posterior papillary muscles are more adversely affected by myocardial ischemia than anterior papillary muscles because of a poor blood supply [6]. In the present case, RVP in addition to the deteriorated hemodynamic state caused by LBBB induced myocardial ischemia in the originally stenosed LCX region followed by the posterior papillary muscle dysfunction. In TAVR candidates, the treatment strategy for coexisting coronary artery lesions, especially non-left anterior descending artery lesions, remains controversial [7]. However, a hypertrophied left ventricle due to AS with a coronary artery lesion is expected to be poorly tolerant with RVP-induced myocardial ischemia. Physicians should recognize that severe MR can be induced by relative myocardial ischemia with papillary muscle dysfunction during TAVR, especially in patients with coexisting coronary artery lesions and an additional factor that deteriorates the hemodynamic state (e.g., LBBB). In this situation, reasonable treatment options should be LV pacing for dyssynchrony, which improves LV synchrony and coaptation of the mitral valve leaflets [8] and can be easily employed by the transapical approach, and IABP support, which increases coronary perfusion and reduces cardiac afterload.

## Conclusions

We have reported a case of acute torrential MR during TAVR caused by LV dyssynchrony and myocardial ischemia, which were clearly detected by TEE and successfully bailed out by LV pacing and IABP support. MR can be seriously exaggerated by various and complicated mechanisms during TAVR. Careful attention should be paid to changes in the degree of MR, which should be rapidly assessed and adequately managed depending on its mechanisms.

## Abbreviations

AS: Aortic stenosis
IABP: Intra-aortic balloon pump
LBBB: Left bundle-branch block
LCX: Left circumflex artery
LV: Left ventricular
MR: Mitral regurgitation
PAP: Pulmonary arterial pressure
RVP: Rapid ventricular pacing
sPAP: Systolic pulmonary arterial pressure
sSAP: Systolic systemic arterial pressure
TAVR: Transcatheter aortic valve replacement
TEE: Transesophageal echocardiography
